# Synergic Effect of Methyl-β-Cyclodextrin and Hydrophilic Polymers on Nepafenac Solubilization: Development of a 0.3% Ophthalmic Solution

**DOI:** 10.3390/molecules30153090

**Published:** 2025-07-23

**Authors:** Maria Grazia Saita, Fabiola Spitaleri, Katia Mangano, Danilo Aleo, Angela Patti

**Affiliations:** 1 MEDIVIS, Via Carnazza 34 C, 95030 Catania, Italy; fabiola.spitaleri@medivis.it (F.S.); danilo.aleo@medivis.it (D.A.); 2 Department of Biomedical and Biotechnological Sciences, University of Catania, Via S. Sofia 89, 95123 Catania, Italy; katia.mangano@unict.it; 3 CNR—Institute of Biomolecular Chemistry, Via Paolo Gaifami 18, 95126 Catania, Italy

**Keywords:** nepafenac, cyclodextrin complex, hydrophilic polymers, ophthalmic solution

## Abstract

Nepafenac is an anti-inflammatory drug used in ophthalmology, marketed as a suspension due to its low aqueous solubility. A solution formulation could provide better bioavailability than suspension and facilitate single unit doses, avoiding the use of preservatives which are required to maintain sterility in multidose packaging. In this study, solubilization of nepafenac was achieved in the presence of randomly methylated β-cyclodextrin (RAMEB) and the actual complexation was assessed by NMR and phase-solubility studies. It was also found that the addition of hydrophilic polymers plays an important role in allowing increased solubilization of nepafenac at the same cyclodextrin concentration. Compared to complexes of nepafenac with other cyclodextrins, only 5% RAMEB was sufficient to solubilize 0.3% (*w*/*v*) nepafenac, enabling for the first time the development of an ophthalmic solution that proved chemically and physically stable for 12 months at 25 °C. The formulated solutions of nepafenac were tested for cytotoxicity on human corneal epithelial cells (HCE-2) and the results suggest their potential as a valuable and safe alternative to the commercially available 0.3% (*w*/*v*) suspension of the drug.

## 1. Introduction

**Scheme 1:**
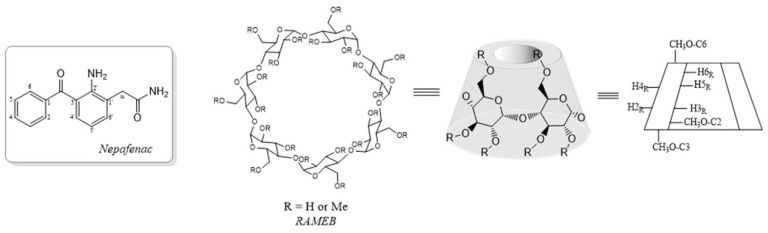
Schematic representation of chemical compounds used in this study.

Nepafenac (2-amino-3-benzoylbenzeneacetamide) (Scheme 1) is a nonsteroidal anti-inflammatory drug (NSAID) approved for the topical treatment of pain and inflammation associated with ocular surgeries, as well as in prevention of cystoid macular edema. Nepafenac specifically inhibits prostaglandin formation in both the anterior and posterior segments of the eye [1]. It is a prodrug that is quickly hydrolyzed by intraocular hydrolases to form the active carboxylic acid, amfenac. The amide group of nepafenac provides superior permeability in ocular tissues (cornea, sclera, and retina/choroid) compared to other topical NSAIDs, such as bromfenac and ketorolac, which are carboxylate salts [2].

Due to its very low solubility in water, nepafenac is commercialized as a 0.1% (*w*/*v*) suspension (Nevanac^®^), with a recommended instillation frequency of three times per day. In 2012, a 0.3% (*w*/*v*) ophthalmic suspension (ILEVRO^®^ 0.3%, Nevanac^®^ 3 mg/mL) was launched on the market and the same efficacy of Nevanac^®^ was demonstrated at reduced daily dose. However, suspension eye drops are uncomfortable causing blurred vision, foreign body sensation, and irritation, leading to increased lacrimation eventually accelerating drainage or spillage of the drug from the eyes. The rapid removal of formulation limits the residence time and hence the bioavailability in the ocular tissues.

Although the topical ophthalmic application of a drug is considered a key route for treating several ocular diseases with good patient compliance, less than 5% of the applied dose reaches the ocular tissues due to the corneal barrier and lacrimation and this problem is even more accentuated in the case of a suspension formulation [3]. Thus, increasing transcorneal permeation and prolonging the contact time of the formulation are major goals to enhance the therapeutic efficacy of ophthalmic drugs.

The use of solution formulations may offer benefits in terms of enhanced permeation, which is proportional to the soluble fraction of drug, and improved patient compliance, for the ease of application and low irritation effects. Hence, enhancing solubility of poorly soluble drugs in water is an active research field and one of the most effective strategies is based on complexation with cyclodextrins [4,5], which are toroidally-shaped cyclic oligomers formed by six to eight D-glucopyranose units bound via 1–4 glycosidic linkage and are characterized by a lipophilic inner cavity and a hydrophilic outer surface. The use of cyclodextrins, which are included among the accepted excipients for drugs [6], as complexing agents has been widely applied to different drugs to improve their solubility and stability in solution [7,8].

For nepafenac, complexation with hydroxypropyl-β-cyclodextrin (HP-β-CD) allowed to solubilize 0.1% (*w*/*v*) drug [9] and the mucoadhesiveness of this solution was subsequently improved by developing an in situ ion-activated gel system based on sodium alginate [10]. Ophthalmic formulations of HP-β-CD/0.1% (*w*/*v*) nepafenac complex, with increased viscosity for the addition of sodium hyaluronate and other suitable excipients, demonstrated higher ex vivo bioavailability than Nevanac^®^ suspension [11].

In a different approach, the self-assembly of cyclodextrins/nepafenac complexes has been exploited for the formation of nanoaggregates or microparticles able to load up to 0.5% (*w*/*v*) nepafenac [12,13] and the scleral permeability as well as anti-inflammatory activity of some formulations of these aggregates containing carboxymethylcellulose were found superior with respect to the reference commercial drug. In addition to cyclodextrins, lipid nanostructured carriers have been also considered to enhance the bioavailability of nepafenac through the formation of hydrogels [14]. However, the high amount of cyclodextrin required for the formation of the above aggregates, the lack of long-term stability data, and technical difficulties in scale-up of hydrogel or nanoaggregate preparations may limit the industrial development of these formulations.

Despite considerable efforts in the field, solution formulations containing more than 0.1% (*w*/*v*) of nepafenac have not yet been reported, and long-term stability data of cyclodextrin-nepafenac complexes are not available. In a previous study, we observed that the HP-β-CD/nepafenac complex prepared according to Shelley et al. [9] suffers some pH-dependent chemical degradation [15] and we planned to evaluate alternative cyclodextrin-based complexes for improving the drug solubility and stability, as a prerequisite for developing a > 0.1% (*w*/*v*) nepafenac ophthalmic solution. Among the different CDs accepted by pharmacopoeia as excipients [6], randomly methylated β-cyclodextrin (RAMEB) has been little used in eye drop formulations [8,16,17,18], even though its higher capacity to solubilize poorly water-soluble compounds compared to other CDs has been highlighted in some cases [19,20,21].

After CD selection by a real-time stability study, this work was focused on the complexation of nepafenac with RAMEB and a crucial role of addition of hydrophilic polymers to the solution for increasing drug solubility up to 0.3% (*w*/*v*) was demonstrated. The feasibility of a solution formulation alternative to the marketed suspension formulation was evaluated and here we report the obtained results.

## 2. Results

### 2.1. Characterization of RAMEB-Nepafenac Complex

At the onset of this study, solutions of nepafenac (0.1% *w*/*v*) and different cyclodextrins were monitored for real-time stability in comparison with the solution of HP-β-CD/nepafenac prepared as reported by Shelley et al. [9]. Quantification of the drug and its possible degradation products was carried out by applying a validated HPLC procedure [15]. Cyclodextrins selected for this study were those accepted by European Medicines Agency (EMA) for ophthalmic use in their non-toxic concentrations [6,22], i.e., HP-β-CD (up to 12.5% *w*/*v*), α-cyclodextrin (≤4% *w*/*v*), sulfobutyl ether-β-cyclodextrin (SBE-β-CD, up to 10% *w*/*v*), and RAMEB (≤5% *w*/*v*). Hyaluronic acid (HA) was employed as a biocompatible viscosity enhancer. While 0.1% (*w*/*v*) nepafenac did not dissolve in the presence of α-cyclodextrin (4% *w*/*v*), clear solutions were obtained with the other tested cyclodextrins.

These solutions were monitored at regular time intervals until the drug content, expressed as a percentage of its initial concentration, remained at or above 90%, a value suitable for pharmaceutical formulations. From the obtained results reported in Table 1, it was evident that formulations with SBE-β-CD were not effective due to the precipitation of an orange solid, which is not merely insoluble nepafenac, but a not identified degradation product. Formulations containing HP-β-CD exhibited limited shelf life at 25 °C, as evidenced by the gradual reduction in drug content over time. Notably, RAMEB-containing solutions showed excellent stability for up to 24 months, even with just 3% (*w*/*v*) cyclodextrin and irrespective of HA presence. On this basis, we decided to assess the RAMEB–nepafenac complexation by NMR and phase solubility studies.

The ^1^H-NMR spectrum of pure RAMEB in D_2_O was quite unresolved, as could be expected by the chemical nature of this commercial cyclodextrin, which is a mixture of at least five compounds in different ratios and with different patterns of substitution resulting in an average of 1.8 methoxyl groups per glucose unit. Although it has been shown that the 2,6-dimethylated isomer (DIMEB) is absent, the chemical structure of the single isomers present in RAMEB has not been assigned [23]. In the ^1^H-NMR spectrum of RAMEB (Figure S1A), distinct broad singlets were observed only for anomeric protons (H-1_R_) at δ 5.16 and 4.95 ppm, attributed to protons adjacent to substituted and unsubstituted hydroxyl groups on C-2. Singlets for methoxyl groups in about a 1:1 ratio were observed at δ 3.28 and 3.45, overlapping the resonances of other protons of the molecule.

In the presence of nepafenac, a slight upfield shift (Δδ = −0.02 ppm) was observed for resonances of anomeric protons (Figure S1B) but it was not possible to detect significant changes in the chemical shifts of diagnostic protons lying in the hydrophobic cavity of RAMEB (H-3_R_ and H-5_R_). In the ^13^C-NMR spectra, a downfield shift was observed for resonances of anomeric carbons (Δδ = +0.16 and +0.18) going from pure RAMEB (Figure S2A) to the RAMEB/nepafenac complex (Figure S2B), while the effects on the other carbon signals were less evident.

The direct comparison between the ^1^H-NMR spectra of pure nepafenac (Figure S3A) and RAMEB–nepafenac (Figure S3B) in D_2_O revealed significant changes in the signal pattern and chemical shifts of all protons of nepafenac following interaction with the cyclodextrin, similar to those reported to occur in the presence of HP-β-CD [9]. Proton H-4′ underwent the more marked variation in its resonance, which appeared at higher field (Δδ = −0.14 ppm), while proton H-6′ was shifted downfield (Δδ = +0.11 ppm).

A more detailed comparison was hampered by the low solubility of nepafenac in D_2_O, but complete assignment of proton and carbon resonances of nepafenac as well as the corresponding changes resulting from the presence of RAMEB (Table S1) were obtained by running the spectra in a D_2_O/*d_6_*-DMSO mixture. In this solvent, the cyclodextrin-induced changes in chemical shifts of nepafenac resonances were confirmed and more clearly distinguished; furthermore, the analysis of ^13^C-NMR spectra revealed marked downfield shifts for the signals of methylene and carbonyl in the -CH_2_CONH_2_ substituent (Δδ = +0.31 and +0.65 ppm, respectively).

**Figure 1:**
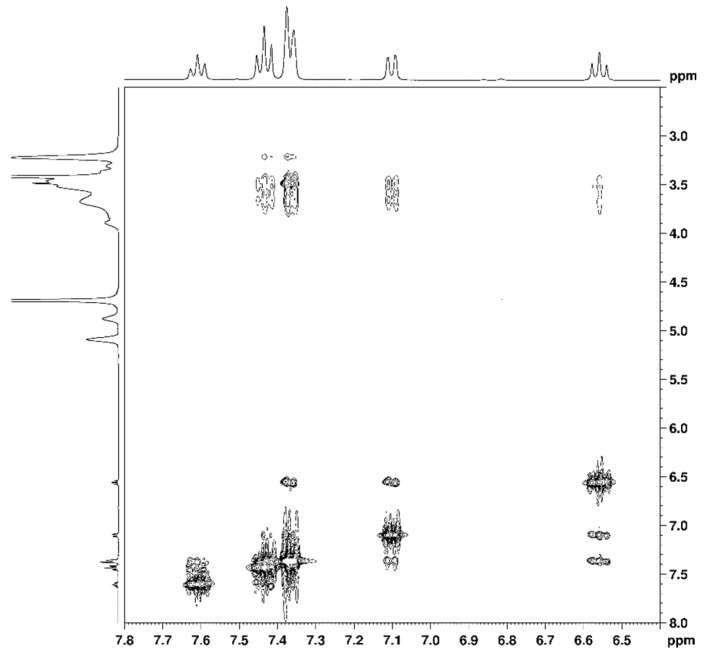
Diagnostic correlations in 2D-ROESY spectrum of RAMEB-nepafenac complex.

The effective complexation of nepafenac was further confirmed by 2D-ROESY spectrum of the RAMEB-nepafenac complex, which shows intermolecular cross-peaks between aromatic protons of nepafenac and cyclodextrin protons (Figure 1). Protons on both the aromatic rings of nepafenac are involved in these intermolecular interactions, suggesting that the molecule is entirely encapsulated in the cyclodextrin cavity.

**Figure 2:**
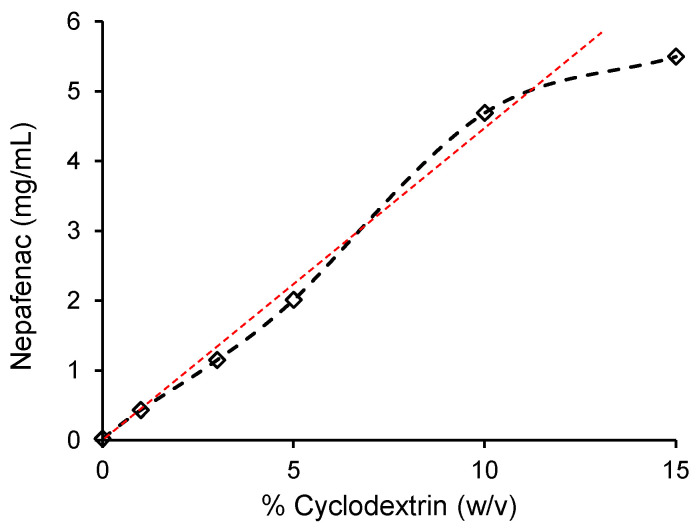
Phase-solubility profile of nepafenac in aqueous solution with RAMEB cyclodextrin.

The phase-solubility profile of nepafenac in an aqueous solution containing RAMEB, shown in Figure 2, clearly demonstrated the increase of drug solubility with increasing host concentration. An AL profile with a slope less than 1 was observed up to 10% (*w*/*v*) concentration of cyclodextrin, suggesting that a 1:1 RAMEB–nepafenac complex is formed. In this range of linearity, an apparent stability constant K1:1 = 8886 (M^−1^) and complexation efficiency CE = 0.873 could be calculated.

A further increase in the concentration of RAMEB resulted in a negative deviation from linearity, probably due to cyclodextrin-induced changes in complex solubility or self-association of cyclodextrin molecules [24].

### 2.2. Effects of Added Hydrophilic Polymers to Solutions of RAMEB-Nepafenac Complex

Based on solubility data, it could be envisioned that a 0.2% (*w*/*v*) nepafenac soluble formulation compatible with EMA recommendations for ophthalmic use could be developed. However, we explored the possibility to further increase the solubility of nepafenac up to 0.3% by adding hydrophilic polymers. It is known that interactions between cyclodextrins/drug complexes and water-soluble polymers often enhance drug solubility, stability, and complexation efficiency, which eventually results in a reduced amount of cyclodextrin needed for drug solubilization [25]. Cyclodextrin-based supramolecular ternary complexes represent an emerging tool in the development of pharmaceutical formulations and the validity of this approach has been confirmed with a large variety of drugs [26,27]. A positive effect of adding hydrophilic polymers on the solubility of nepafenac had been previously observed in formulations containing micro- and nanoparticles aggregates formed between complexes of nepafenac and two different cyclodextrins [12,13].

To assess a possible enhancement of nepafenac solubility, three hydrophilic polymers, PVA, PVP, or HPMC, in two different concentrations (0.5% or 1.0% *w*/*v*) were added to a suspension of RAMEB (5% *w*/*v*) and nepafenac (0.3% *w*/*v*) in phosphate buffer at pH 7.6, chosen as optimal pH for long-term stability of the drug [15], and a reference sample without any added polymer (S1) was also prepared.

All the samples (see Table 2 for composition) were heated at 60 °C for 5 h and under these conditions, which should promote the formation of ternary complexes [26], clear solutions were obtained, then left to stand at 25 °C for visual monitoring.

**Figure 3:**
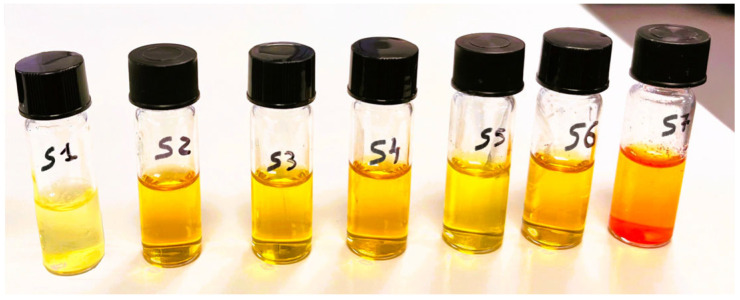
Physical appearance of samples S2–S7 (see Table 2) after stress study.

As could be expected, a yellow precipitate of nepafenac was observed within 24 h at 25 °C in the sample containing only the cyclodextrin complex (S1), while all the other samples (Table 2) containing a polymer additive remained as solutions for several days. A stress study was carried out on samples S1–S7 and some changes in the solution appearance (Figure 3) along with a decrease in nepafenac content were observed, particularly in those containing HPMC (Table 2).

These effects were more pronounced at higher HPMC concentrations, with sample S7 showing some decrease in the pH value and the presence of an orange precipitate at the final time point. On the contrary, better stability of nepafenac was observed in the presence of 1.0% (*w*/*v*) PVP or PVA compared to the corresponding solutions containing a lower amount of the polymers. These data confirm the high stability of the RAMEB-nepafenac complex per se and highlight the crucial role of the added polymers in improving its solubility.

It is known that cyclodextrins in aqueous solution can assembly in self-organized aggregates (200–300 nm), the formation of which is driven by hydrogen bonds between molecules and increases with concentration. Modified cyclodextrins display a reduced tendency to self-clustering due to the substitution of hydroxyl groups present in native cyclodextrins with less polar hydrocarbon groups, thereby resulting in a decreased hydrogen bonding ability. Cyclodextrin–guest complexation can enhance the tendency to aggregation, leading to larger aggregates, in some case with micelle-like structure [27].

The obtained stable solutions (samples S2–S5, Table 2) were then analyzed by dynamic light scattering (DLS) in comparison with the corresponding solutions without nepafenac (Table 3) to evaluate the effect of the added hydrophilic polymer on the aggregation of RAMEB and RAMEB-nepafenac complex.

In a 5% solution of RAMEB, two almost equivalent populations of cyclodextrin monomers (1.83 ± 0.01 nm) and nanoaggregates (212.2 ± 5.86 nm) were detected, but in the presence of added PVP, in both 0.5% (*w*/*v*) and 1% (*w*/*v*) concentration, aggregates with reduced size (27.38 ± 2.09 and 18.22 ± 1.5 nm, respectively) became predominant (compare entries 2 and 4 with entry 1 in Table 3), so confirming the ability of hydrophilic polymers to intercalate between cyclodextrin molecules [28].

The aggregation tendency was not altered by the complexation of RAMEB with nepafenac and solutions S2 and S3 exhibited particle size distributions quite similar to those observed without the drug, with S2 showing a lower polydispersity index (PDI) than S3. Reduced aggregation tendency of RAMEB was also observed in the presence of PVA at both 0.5% and 1% (*w*/*w*) concentrations (compare entries 1, 6, and 8 in Table 3) and solutions S4 and S5, containing the RAMEB-nepafenac complex, showed a closely comparable particle distribution (compare entries 6 and 7 or 8 and 9 in Table 3).

The observed data indicated that hydrophilic polymers PVP or PVA, that share similarity in their vinyl-derived backbone but differ for side groups involved in hydrogen bonds with water/cyclodextrin molecules, showed the same efficiency in decreasing the self-aggregation tendency of both RAMEB and its nepafenac complex.

All the tested solutions S2–S5 showed a PDI index slightly superior to that considered as indicative of a uniform particle distribution (PDI < 0.3) [29], except for S2, and negative zeta potential (Z) values compatible with a stable dispersion system and in agreement with reported values for other ternary nepafenac-cyclodextrin-polymer complexes with PVA or PVP [13]. The presence of the water-soluble polymer seems to favor steric repulsion, as the absolute value of Z increases with the polymer concentration and more negative values were measured for PVA-containing formulations compared to those with PVP.

### 2.3. Development of 0.3% Nepafenac Solution Formulation and Preliminary Biological Studies

On the basis of their superior stability (see Table 2) and acceptable homogeneity in particle size, S3 and S5 platforms were then formulated by the addition of 0.1% (*w*/*v*) hyaluronic acid as viscosifying agent, 0.9% (*w*/*v*) glycerol and 0.2% (*w*/*v*) NaCl as osmotic agents and 0.02% (*w*/*v*) EDTA-Na_2_ as stabilizer agent. These ophthalmic preparations were filtered through sterile 0.2 μm filters and monitored for real-time stability according to ICH guidelines at 25 °C ± 2 °C and R.H. 60% ± 5% [30].

Both the obtained formulations, labeled as F3 and F5 to designate the solution containing the nepafenac-RAMEB complex suitable for ophthalmic use, were found to be chemically stable, with a decrease in nepafenac content less than 2% over 12 months (Table S2), and maintained their physical state in the same time period. DLS measurements on formulations F3 and F5 revealed the presence of some larger aggregates (>200 nm) compared to the parent solutions of the nepafenac-RAMEB complex S3 and S5, the formation of which is probably promoted by the presence of the excipients used. Both formulations, however, were found to be dimensionally stable after 12 months (Table S3) without any evidence of further aggregation phenomena.

Compared to other reported cyclodextrin–nepafenac systems [9,10,11,12,13], the developed formulations offer advantages in: (a) solubilizing 0.3% (*w*/*v*) nepafenac; (b) requiring a smaller amount of cyclodextrin for complexation; (c) demonstrating long-term stability; and (d) having a physical state compatible with sterilization by simple filtration of the bulk formulation. Point (d) is particularly important in manufacturing since obtaining a sterile pharmaceutical suspension is a technically challenging task.

Furthermore, the availability of soluble nepafenac formulations could meet the growing demand for preservative-free eye drops because they can be marketed in single-dose containers or in the innovative multidose preservative-free packaging [31], equipped with a sterilizing filter (0.2 μm) to ensure sterility once opened. Instead, these types of packaging are not compatible with nepafenac suspensions, for which the only option is a standard multi-dose packaging whose sterility once opened is maintained by adding a preservative in the formulation. The marketed suspension of nepafenac, in fact, contains benzalkonium chloride as a preservative, which, however, is associated with adverse effects during therapy [32,33].

Cytotoxicity of formulations F3 and F5 was evaluated on a human corneal epithelial cell line (HCE-2) in comparison with the marketed formulation at the same 0.3% (*w*/*v*) concentration of the drug, that is available only as suspension (Nevanac^®^ 3 mg/mL)

**Figure 4:**
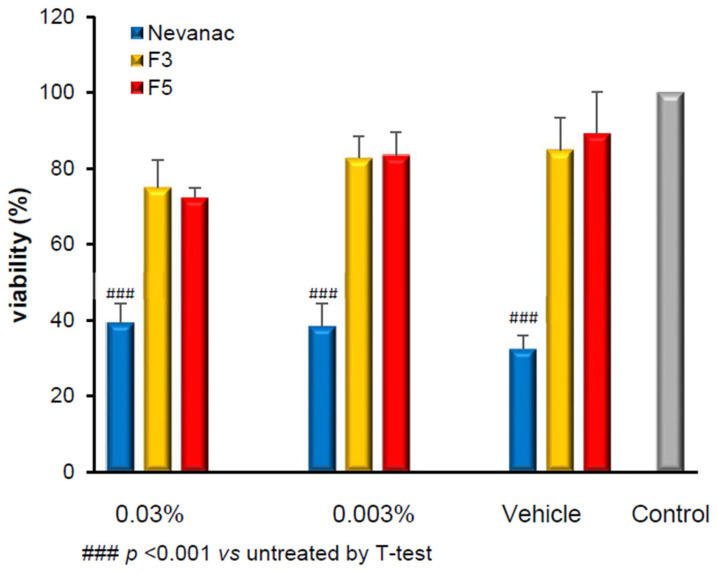
Effects of Nevanac^®^ 3 mg/mL and formulations F3 and F5 on HCE-2 cells viability after 24 h of incubation by MTT test. Data are expressed as percentage of viability compared to untreated cells ± RSD, n = 3 for treatment.

The results of the cytotoxicity test, carried out at two different dilutions (1:10 and 1:100), indicated that solution formulations F3 and F5 of nepafenac with cyclodextrin–hydrophilic polymers had better cell viability than the suspension formulation. In particular, the treatment with Nevanac^®^ 3 mg/mL as well as its vehicle, induced a significant reduction in cell viability compared to untreated cells; on the other hand, the cell viability after treatment with F3 and F5 was almost two-fold higher than the corresponding dosage of Nevanac^®^ 3 mg/mL and even the related vehicles were well-tolerated compared to untreated cells (Figure 4).

Cytotoxicity of Nevanac^®^ 3 mg/mL is associated with the vehicle rather than the drug, probably due to the preservative compound benzalkonium chloride, whose destructive effects through different mechanisms on corneal epithelial cells have been proven [34,35].

The observed low toxicity of F3 and F5 is a significant finding, as reduced toxicity is crucial for ocular medications, particularly when treating conditions such as post-cataract surgery inflammation.

## 3. Materials and Methods

### 3.1. General

Nepafenac was obtained from MicroLabs Ltd., Bangalore, India. RAMEB (DS 12.0, MW 1310 Da) was supplied by Wacker Chemie AG, sulfobutyl ether-β-cyclodextrin (SBE-CD, D.S. 6.5, MW 2163 Da) by CicloLab Ltd., α-CD and HP-β-CD (DS 6.9, MW 1537 Da) by Roquette Italia, Alessandria, Italy. Polyvinyl alcohol polymer (PVA, Aver. MW 145 KDa) was obtained from Merck (Milan, Italy), whereas Polyvinylpirrolydone (PVP, Aver. MW 58 KDa) and hydroxypropylmethylcellulose (HPMC, Aver. MW 400 KDa) were given by Ashland, Wilmington, DE, USA. Sodium hyaluronate (HA, Aver. MW 1.64 MDa) was supplied by Contipro, Dolní Dobrouč, Czech Republic. Commercial Nevanac^®^ 3 mg/mL eye drop (Alcon Inc., Fort Worth, TX, USA) was purchased in the local pharmacy (LOT 22C10CB). Vehicle of Nevanac^®^ 3 mg/mL was prepared according to patented data [36]. All used excipients were pharma grade. Milli-Q water, previously filtered through 0.45 μm cellulose acetate filter, was used for all the aqueous solutions.

^1^H- and ^13^C-NMR spectra were recorded on a Bruker Avance™ 400 (Bruker Italia, Milan, Italy) instrument at 400.13 and 100.03 MHz, respectively, and chemical shifts (δ) are relative to the residual solvent peak. The 2D-experiments were carried out using an inverse multinuclear probe with pulse-field Z-gradient and standard Bruker pulse sequence programs (TOPSPIN 2.1, avance-version). Osmolality was measured with Osmomat 030-GONOTEC instrument (Gonotech GmbH, Berlin, Germany).

### 3.2. HPLC Analyses

HPLC analyses were carried out on an Agilent 1260 Infinity (Agilent Technologies, Santa Clara, CA, USA) instrument equipped with a refrigerated autosampler, thermostatted column compartment, and UV-vis detector connected with OpenLab CDS 3.5.0 software. Chromatographic analyses of nepafenac were performed by using an EVO Kinetex (Phenomenex) column (150 mm × 4.6 mm i.d.) isocratically eluted at 25 °C and flow of 0.7 mL/min with a 35:65 (*v*/*v*) mixture of CH_3_CN and 20 mM ammonium formate buffer at pH 6.0, following a previously validated method [15]. The linear correlation coefficient for the calibration curve was 0.9999. Statistical evaluation of the data was performed using one-way analysis of variance (ANOVA). The Bonferroni multiple comparison test was used to compare the significance of the difference between the groups and a *p*-value ≤ 0.05 was accepted as significant.

### 3.3. Real-Time Stability of Solutions Containing 0.1% Nepafenac and Different Cyclodextrins

Nepafenac solution at 0.1% (*w*/*v*) in different cyclodextrins were formulated in the appropriate aqueous ophthalmic vehicle (see Table 1) at physiological pH. Solutions were sterilized by filtration through 0.2 μm sterile cellulose acetate filters and then dispensed in polypropylene sterile vials (2 mL) under aseptic conditions and stored at room temperature (25 ± 2 °C; R.H. 60 ± 5%) for up to 24 months. At intervals of three months the content of nepafenac in the solutions was evaluated by HPLC on three independent samples obtained by 1:20 dilution with distilled water.

### 3.4. Phase-Solubility Study

Following a reported procedure [12], an excess of nepafenac (50 mg) was added to an aqueous solution (5 mL) of increasing concentrations of RAMEB (1–15% *w*/*v*). The suspensions were sealed in amber glass vials and subjected to sonication in a heated bath at 60 °C for 1 h, then cooled to room temperature and stirred magnetically for one week at 25 ± 2 °C. Afterward, the suspensions were centrifuged for 10 min at 25 °C at 4670× *g*. The resulting supernatants were filtered on a 0.45 µm cellulose acetate syringe-filter to remove any undissolved nepafenac particles. The filtered samples were then analyzed using the validated method. Phase solubility diagrams were created by correlating the amount of dissolved nepafenac with cyclodextrin content.

### 3.5. Preparation of Nepafenac/RAMEB/Hydrophilic Polymers Complexes

The influence of hydrophilic polymers on the solubilization of nepafenac at 0.3% *w*/*v* by RAMEB was investigated in buffered phosphate solutions at pH 7.6 containing 5% *w*/*v* RAMEB and 0.5% or 1% (*w*/*v*) of HPMC, PVP or PVA (samples S2–S7 in Table 2). In detail, nepafenac (30 mg) was added to 10 mL of each solution and the resulting suspensions were stirred in a heated bath at 60 °C for 5 h, then cooled to room temperature. The clear yellow solutions were filtered on 0.2 μm syringe-filters under aseptic condition and stored in a sterile glass vial.

### 3.6. Stress Study

The stress study was carried out at 60 °C for 15 days on samples S1–S7. Aliquots of samples (2 mL) were withdrawn every week and stored at −16 °C until quantification of nepafenac by HPLC.

### 3.7. Dynamic Light Scattering (DLS) and Zeta Potential Measurements

DLS measurements (size, polydispersity index, intensity distributions) and zeta potentials were carried out on a Zetasizer Nano Pro instrument (Malvern instrument Ltd., Malvern, Worcestershire, UK). All the solutions, whose composition is reported in Table 3, were prepared in 10 mM phosphate buffer pH 7.6 and filtered through a 0.2 μm sterile filter before analysis. Measurements were performed in triplicate at 25 °C with a scattering angle of 173°, after allowing the solutions to equilibrate for 15 min.

### 3.8. Formulation of Ophthalmic Solutions Containing 0.3% (w/v) Nepafenac

In a typical procedure for the preparation of formulations F3 and F5, 2.5 g of RAMEB and 10 mL of 5% PVP or 5% PVA solution were added to 40 mL of distilled water (polymer amount 1% *w*/*v* in the final solution). Nepafenac (150 mg) was added to this solution and the resulting suspension was stirred in a heated bath at 60 °C for about 5 h until complete solubilization of nepafenac. The obtained solution was cooled to room temperature and glycerol (450 mg), NaCl (100 mg), EDTA-Na_2_ (10 mg), sodium phosphate bibasic dodecahydrate (180 mg), and hyaluronic acid (50 mg) were added until complete homogenization of HA. Then, the clear yellow solution was filtered on 0.2 μm syringe-filters under aseptic condition and stored in a sterile polypropylene vial.

### 3.9. Cell Line

HCE-2 (ATCC CRL-11135) Human Corneal Epithelial cell line was purchased from American Type Culture Collection (Manassas, VA, USA). HCE-2 cells were cultured in keratinocyte serum-free medium (KSFM) (Gibco, Thermo Fisher Scientific, Monza, Italy) supplemented with 0.05 mg/mL bovine pituitary extract (BPE) (Gibco), 500 ng/mL hydrocortisone (Gibco), 0.005 mg/mL insulin (Gibco), and 5 ng/mL epidermal growth factor (EGF) (Gibco). Cells were maintained at 37 °C and 5% CO_2_ in a cell culture incubator and used within 10 passages after thawing.

### 3.10. Cytotoxic Assay

MTT (3-(4,5-Dimethylthiazol-2-yl)-2,5-diphenyl tetrazolium bromide (Sigma-Aldrich, Milan, Italy) assay was carried out to evaluate the biocompatibility of formulations F3 and F5 in comparison with the commercial suspension of nepafenac 0.3%, at different dilutions, in HCE-2 cells. HCE-2, 5000 cells/well, were plated in 200 μL of complete medium and incubated at 37 °C and 5% CO_2_ in a cell culture incubator. At 80% confluence, the medium (KSFM) in the well plate was discarded, the cells were rinsed with phosphate buffer, and then 200 μL of medium containing F3, F5, or marketed Nevanac^®^ 0.3%, at concentrations of 0.03% and 0.003% as well as their respective vehicles were added to the cells, each in triplicate. Following 24 h of incubation, cells were assessed for their viability by adding 0.5 mg/mL MTT in PBS per well and the plate was further incubated at 37 °C for 3 h. Thereafter, the medium was removed and 100 μL of DMSO was added to each well to dissolve insoluble purple formazan crystals. The optical density was quantified at 570 nm using spectrophotometric analysis (Tecan Sunrise Microplate Reader). The results were expressed as mean ± RSD (n = 3 experiments).

## 4. Conclusions

In this study, the long-term stability of complexes of nepafenac (0.1% *w*/*v*) with different cyclodextrin was compared with the aim to develop a stable, aqueous eye drop formulation, that could contribute to overcoming critical issues associated with the commercial suspension formulation of the drug. The best results were obtained in the presence of randomly methylated-β-cyclodextrin and its complexation with nepafenac was confirmed by NMR and phase-solubility study. By suitable addition of hydrophilic polymers that promote the formation of ternary complexes, an increase in solubilized drug up to 0.3% (*w*/*v*) concentration was achieved and the resulting solutions were analyzed for their size distribution and stability. The best soluble platforms were formulated for ophthalmic use and tested for cytotoxicity, revealing reduced toxicity compared to the marketed suspension product. Handling solution formulations of nepafenac could facilitate manufacturing and enable the development of preservative-free eye drops compatible with modern multidose packaging. Further studies are currently in progress to scale-up process and evaluate the efficacy of the solution formulation in vivo, especially considering that solution-based eye drops containing cyclodextrins like RAMEB can enhance epithelial permeability and improve ocular bioavailability compared to suspensions.

## Figures and Tables

**Table 1:** Composition of nepafenac-containing solutions *^a^* and variation of the drug content with time *^b^*.

Solution	Cyclodextrin (*% w*/*v*)	Additive (*% w*/*v*) *^c^*	Months						
			*1*	*3*	*6*	*9*	*12*	*18*	*24*
**Reference**	**HP-β-CD (6.0)**	HA (0.10)	97	92	84	-- *^d^*	--	--	--
**1**	HP-β-CD (4.0)	HA (0.15)	96	91	80	-- *^d^*	--	--	--
**2**	RAMEB (4.0)	HA (0.10)	100	99	98	97	96	95	94
**3**	RAMEB (3.0)	--	100	98	97	96	95	93	92
**4**	SBE-β-CD (6.0)	HA (0.10)	97	90	ppt *^e^*	--	--	--	--
**5**	SBE-β-CD (4.0)	HA (0.10)	96	ppt *^e^*	--	--	--	--	--

*^a^* In 10 mM phosphate buffer pH 7.5; *^b^* The solutions were maintained at 25 °C and drug content was expressed as % of the initial concentration; *^c^* Glycerol (0.9% *w*/*v*), mannitol (1.0% *w*/*v*) and EDTA-Na_2_ (0.02% *w*/*v*) were also present in the solutions as excipients; *^d^* Not analyzed; *^e^* A precipitate was formed in the solution.

**Table 2:** Composition *^a^* and stability *^b^* of solutions of RAMEB-nepafenac complex in the presence of hydrophilic polymers.

Sample Code	Polymer (% *w*/*v*)	Nepafenac % *^c^*	pH	Presence of Solid
S1	none	97.2	7.5	- *^d^*
S2	PVP (0.5)	97.3	7.5	–
S3	PVP (1.0)	98.6	7.5	–
S4	PVA (0.5)	97.7	7.5	–
S5	PVA (1.0)	98.3	7.5	–
S6	HPMC (0.5)	96.3	7.4	–
S7	HPMC (1.0)	95.2	7.2	+

*^a^* RAMEB (5% *w*/*v*), nepafenac (0.3% *w*/*v*) and polymer (0.5 or 1.0% *w*/*v*) dissolved in 10 mM phosphate buffer pH 7.6 (10 mL). *^b^* Solutions were maintained at 60 °C and analyzed after 15 days. *^c^* Expressed as percentage of the initial content of nepafenac. *^d^* Nepafenac precipitates on cooling to room temperature.

**Table 3:** Mean values of hydrodynamic radius, peak intensity (I%), polydispersity index (PDI), and zeta potential of RAMEB/polymers mixtures and RAMEB/nepafenac complexes in samples S2–S5 *^a^*.

Entry	Sample Composition	D_H_ (nm)	I %	PDI	Z (mV)
1	5% RAMEB *^b^*	1.83 ± 0.01	54.82 ± 1.26	0.56 ± 0.03	−19.67 ± 1.54
		212.2 ± 5.86	44.2 ± 0.98		
2	0.5% PVP/5%RAMEB *^b^*	2.35 ± 0.29	20.7 ± 1.95	0.56 ± 0.01	−9.57 ± 0.10
		27.38 ± 2.09	78.51 ± 3.07		
3	S2	2.28 ± 0.11	26.28 ± 0.42	0.14 ± 0.01	−12.34 ± 3.64
		25.71 ± 2.75	73.72 ± 0.42		
4	1% PVP/5%RAMEB *^b^*	2.54 ± 0.05	7.16 ± 0.61	0.30 ± 0.03	−11.38 ± 1.19
		18.22 ± 1.50	92.96 ± 3.66		
5	S3	2.6 ± 0.32	10.7 ± 1.68	0.34 ± 0.05	−15.25 ± 3.78
		19.34 ± 0.66	88.5 ± 1.83		
6	0.5% PVA/5%RAMEB *^b^*	1.98 ± 0.11	13.7 ± 0.54	0.48 ± 0.01	−12.82 ± 3.00
		23.77 ± 0.58	84.0 ± 0.40		
7	S4	2.36 ± 0.05	25.77 ± 0.40	0.54 ± 0.01	−17.78 ± 0.83
		25.19 ± 0.89	71.77 ± 0.56		
8	1% PVA/5%RAMEB *^b^*	1.78 ± 0.12	5.6 ± 0.35	0.28 ± 0.03	−7.28 ± 1.29
		27.8 ± 1.37	92.8 ± 1.14		
9	S5	1.93 ± 0.15	7.5 ± 0.53	0.40 ± 0.02	−19.56 ± 3.84
		28.84 ± 0.52	87.86 ± 0.56		

*^a^* Composition of samples S2–S5 as in Table 2; *^b^* In 10 mM phosphate buffer pH 7.6 (10 mL), % expressed as *w*/*v*.

## Data Availability

The original contributions presented in this study are included in the article/Supplementary Material. Further inquiries can be directed to the corresponding authors.

## Supplementary Materials

The following supporting information can be downloaded at: https://www.mdpi.com/article/10.3390/molecules30153090/s1, NMR data (^1^H- and ^13^C-NMR spectra) of nepafenac and RAMEB cyclodextrin/nepafenac complex and assignment of their resonances; DLS parameters of drug ophthalmic formulations; real time monitoring of properties of 0.3% nepafenac ophthalmic formulations F3 and F5 over the time.
