# Supplementary material for: Synergic Effect of Methyl-β-Cyclodextrin and Hydrophilic Polymers on Nepafenac Solubilization: Development of a 0.3% Ophthalmic Solution
Source: Molecules. 2025 Jul 23;30(15):3090. doi: 10.3390/molecules30153090 (PMC12348884; doi:10.3390/molecules30153090)
Supplement: Supplementary file 1 [file molecules-30-03090-s001.zip › molecules-3745972-supplementary.pdf]

Synergic effect of methyl-β-cyclodextrin and hydrophilic polymers on nepafenac solubilization: development of a 0.3% ophthalmic solution

Maria Grazia Saita,[1*] Fabiola Spitaleri,[1] Katia Mangano,[2] Danilo Aleo[1] and Angela Patti[3*]

[1]MEDIVIS – Via Carnazza 34 C, I- 95030 Tremestieri Etneo (Catania)

[2]Department of Biomedical and Biotechnological Sciences, University of Catania, Via S. Sofia 89, 95123 Catania, Italy

[3]CNR – Institute of Biomolecular Chemistry, Via Paolo Gaifami 18, I-95126 Catania, Italy

[*]Correspondence: mariagrazia.saita@medivis.it; angela.patti@cnr.it

Supporting information

	Page
**Figure SI-1.** ^{1}H-NMR spectra of RAMEB[#] and RAMEB/nepafenac complex in D_2O	SI-1
**Figure SI-2.** ^{13}C-NMR spectra of nepafenac and RAMEB/nepafenac complex in D_2O	SI-1
**Figure SI-3.** ^{1}H-NMR spectra of nepafenac and RAMEB/nepafenac complex in D_2O	SI-2
**Table SI-1.** Changes of NMR chemical shifts of nepafenac upon complexation with RAMEB	SI-3
**Table SI-2.** Real time monitoring of properties of 0.3% nepafenac ophthalmic formulations F3 and F5 over the time	SI-4
**Table SI-3.** DLS parameters of formulations F3 and F5	SI-4

[#]RAMEB: randomly methylated β-cyclodextrin

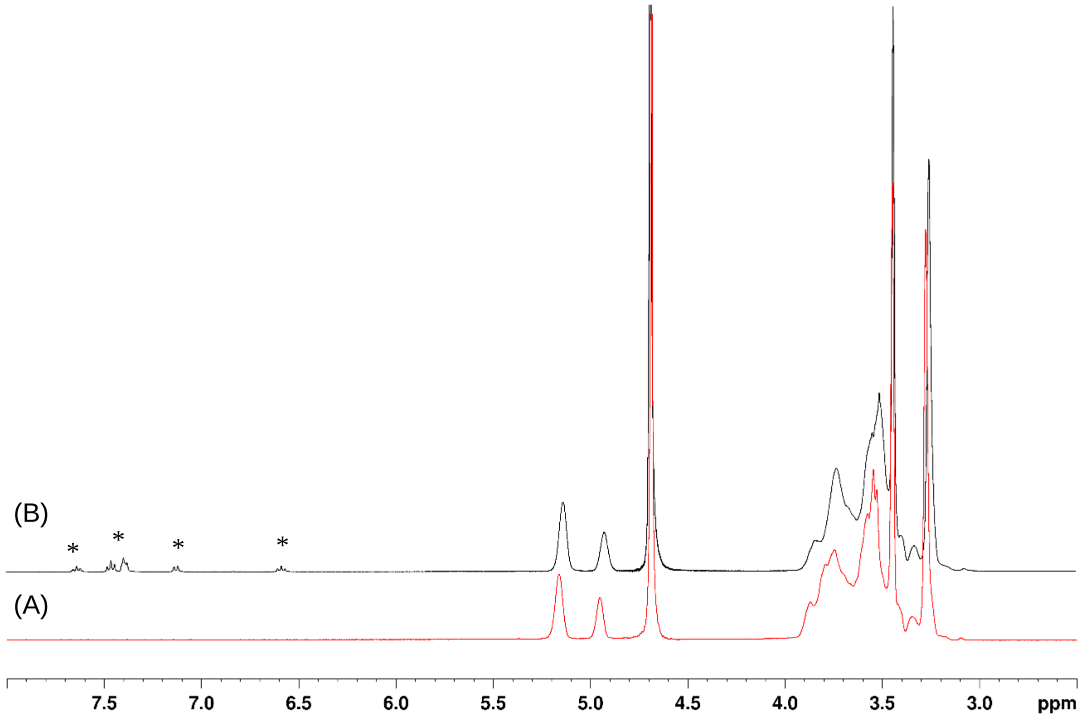

* = resonances of nepafenac

Figure SI-1. ^{1}H-NMR spectra of (A) RAMEB (red trace) and (B) RAMEB/nepafenac complex (black trace) in D_2O

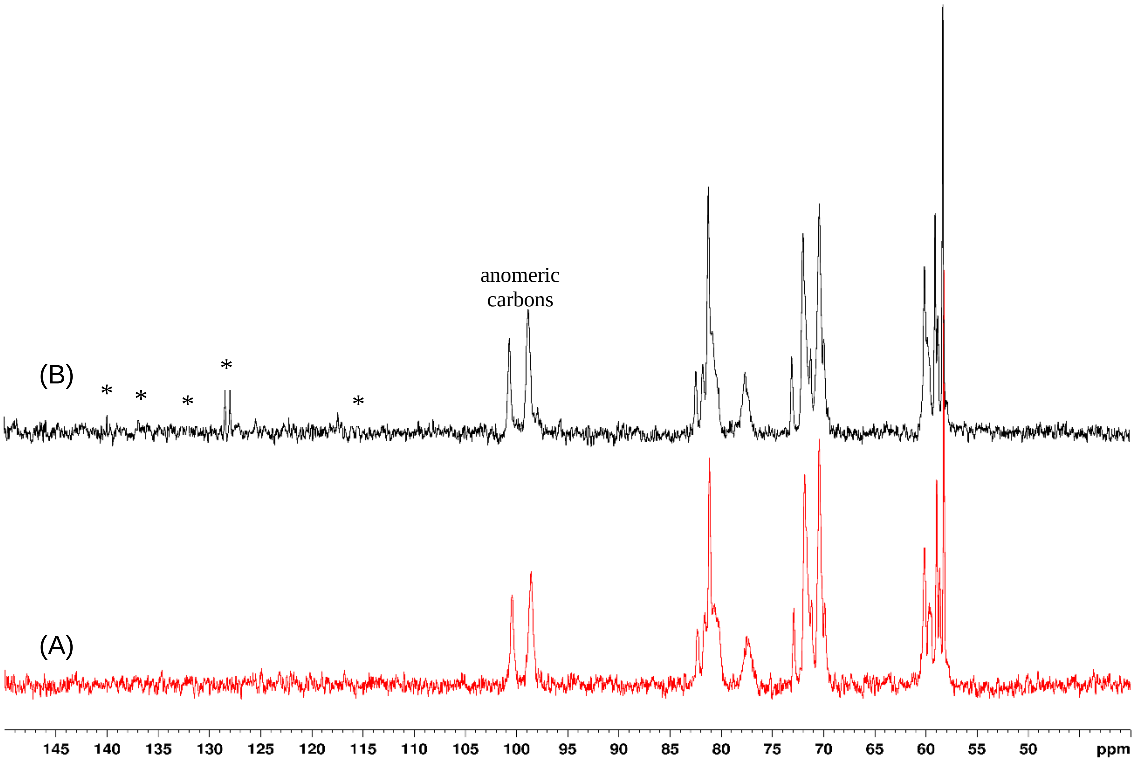

* = resonances of nepafenac

Figure SI-2. ^{13}C-NMR spectra of (A) RAMEB (red trace) and (B) RAMEB/nepafenac complex (black trace) in D_2O

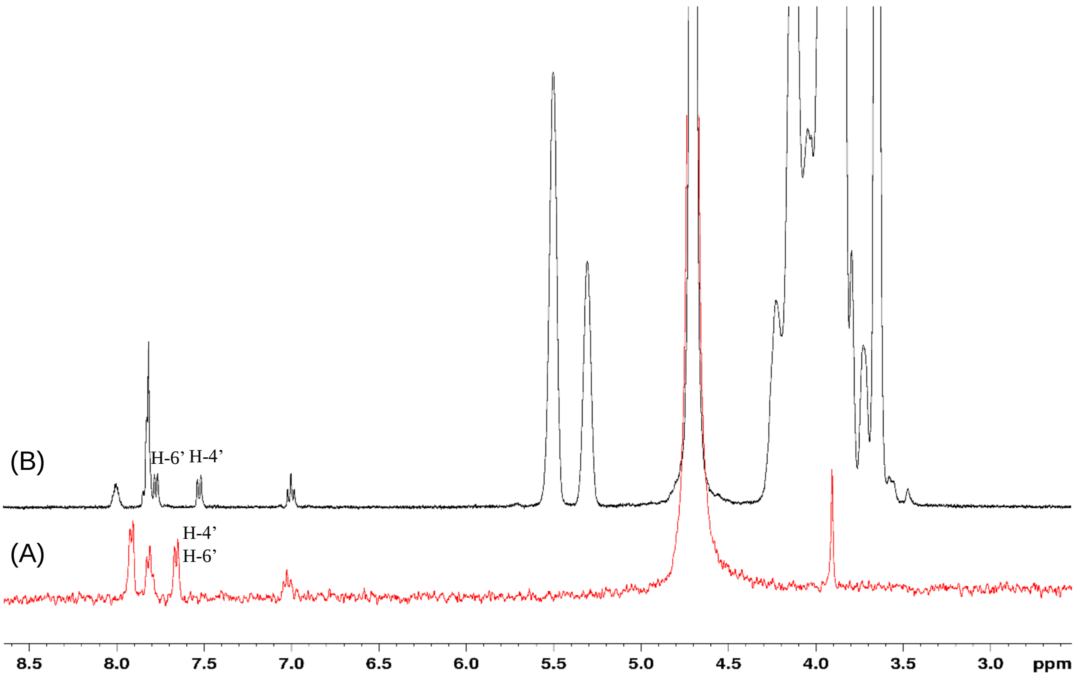

Figure SI-3. ^{1}H-NMR spectra of (A) nepafenac (red trace) and (B) RAMEB/nepafenac complex (black trace) in D_2O (45 °C)

Table SI-1. Changes of NMR chemical shifts of nepafenac upon complexation with RAMEB[a]

	Nepafenac		Nepafenac/RAMEB Complex		Δδ	
**Position**	^{1}H-NMR (δ)	^{13}C-NMR (δ)	^{1}H-NMR (δ)	^{13}C-NMR (δ)	^{1}H-NMR	^{13}C-NMR
$\underline{CH_2}CONH_2$	3.409	40.34[b]	Nd[c]	40.65[b]	--	+0.31
5’	6.568	117.63	6.549	117.88	- 0.019	+0.25
4’	7.215	135.91	7.168	135.77	- 0.047	-0.14
6’	7.264	138.80	7.280	138.99	+0.016	+0.19
1’		124.32		124.51		+0.19
3’		120.08		120.25		+0.17
2’		151.88		152.17		+0.29
1		141.94		142.20		+0.26
4	7.43-7.57	130.88 x 2	7.38-7.46	130.88		--
3,5		131.13 x 2		131.10		-0.03
2,6		134.09		134.30		+0.21
CONH$_a$		176.55		177.20		+0.65
CO		202.04		202.00		-0.04

[a] ^{1}H- and ^{13}C-NMR spectra were registered in D_2O/d_6-DMSO 1:1 at 400.13 and 100.03 MHz, respectively.[b]Identified by DEPT-135 experiment. [c]Overlapped by cyclodextrin resonances.

Table SI-2. Real time monitoring of properties of 0.3% nepafenac ophthalmic formulations F3 and F5 over the time[a]

**Formulation**[b]	**Parameter**	**Time** *(months)*					
		0	*1*	*3*	*6*	*9*	*12*
**F3**	Title%	100	100	99.5	99.0	98.5	98.1
	pH	7.6	7.6	7.6	7.6	7.5	7.5
	Osm[c]	273	275	275	283	283	285
**F5**	Title%	100	100	99.7	99.4	99.4	98.5
	pH	7.6	7.6	7.6	7.6	7.5	7.5
	Osm[c]	270	270	270	275	281	281

[a]Formulations were maintained at 25±2 °C and 60±5% R.H.; [b]Formulations contained nepafenac (0.3% w/v), RAMEB (5.0% w/v), polymer PVA or PVP (1% w/v), sodium hyaluronate (0.1%), glycerol (0.9% w/v), NaCl (0.2 % w/v), EDTA-Na_2 (0.02% w/v), 10 mM phosphate buffer pH 7.6. [c]Expressed in mOsmol/kg

Table SI-3. DLS parameters of formulations F3 and F5[a]

**Time (months)**[b]	**Formulation**	**D_H (nm)**	**I %**	**PDI**	**Z (mV)**
0	F3	2.06±0.28<br>21.93±1.68<br>262.3±28.3	9.11±0.80<br>72.5±2.02<br>18.4±1.80	0.44±0.02	-10.3±1.28
12	F3	2.07±0.22<br>22.68±0.14<br>268.5±39.13	12.69±0.42<br>67.30±1.86<br>16.49±1.35	0.66±0.03	-15.3±0.41
0	F5	1.80±0.1<br>25.3±1.08<br>226.9±32.8	5.7±0.20<br>39.9±2.17<br>51.8±2.60	0.57±0.26	-20.0±1.06
12	F5	24.52±0.65<br>200.9±5.26	42.89±1.18<br>57.11±1.18	0.58±0.01	-25.7±1.50

[a]Formulations were prepared with nepafenac (0.3% w/v), RAMEB (5.0% w/v), polymer PVA or PVP (1% w/v), sodium hyaluronate (0.1%), glycerol (0.9% w/v), NaCl (0.2 % w/v), EDTA-Na_2 (0.02% w/v), 10 mM phosphate buffer pH 7.6 and filtered through sterile 0.2 μm filters. [b]The formulations were stored in a sterile polypropylene vial and maintained at 25 °C.
